# PQN-75 is expressed in the pharyngeal gland cells of *Caenorhabditis* *elegans* and is dispensable for germline development

**DOI:** 10.1242/bio.027987

**Published:** 2017-09-15

**Authors:** Jesse D. Rochester, Paige C. Tanner, Catherine S. Sharp, Karolina M. Andralojc, Dustin L. Updike

**Affiliations:** The Mount Desert Island Biological Laboratory, Bar Harbor, ME 04672, USA

**Keywords:** *C. elegans*, Pharynx, PQN-75, FG-repeat, Pharyngeal gland cells

## Abstract

In *Caenorhabditis elegans*, five pharyngeal gland cells reside in the terminal bulb of the pharynx and extend anterior processes to five contact points in the pharyngeal lumen. Pharyngeal gland cells secrete mucin-like proteins thought to facilitate digestion, hatching, molting and assembly of the surface coat of the cuticle, but supporting evidence has been sparse. Here we show pharyngeal gland cell expression of PQN-75, a unique protein containing an N-terminal signal peptide, nucleoporin (Nup)-like phenylalanine/glycine (FG) repeats, and an extensive polyproline repeat domain with similarities to human basic salivary proline-rich pre-protein PRB2. Imaging of C-terminal tagged PQN-75 shows localization throughout pharyngeal gland cell processes but not the pharyngeal lumen; instead, aggregates of PQN-75 are occasionally found throughout the pharynx, suggesting secretion from pharyngeal gland cells into the surrounding pharyngeal muscle. PQN-75 does not affect fertility and brood size in *C. elegans* but confers some degree of stress resistance and thermotolerance through unknown mechanisms.

## Abstract (precis)

**Summary:** PQN-75 is expressed in pharyngeal gland cells and shares similarity with human basic salivary proline-rich protein PBR2, suggesting evolutionary conservation between gland cells in the upper digestive tract.

## INTRODUCTION

Phenylalanine/glycine (FG) repeats create intrinsically disordered protein domains and are common in the nuclear pore complex (NPC) where FG-nucleoporin (Nups) constitute the permeability barrier of the pore and facilitate transport between the nucleus and cytoplasm (reviewed in Beck and Hurt, 2016). In *Caenorhabditis elegans*, FG-repeat domains are also found outside the periphery of NPCs in the core P-granule proteins GLH-1, GLH-2, GLH-4, DDX-19, and RDE-12 (Sheth et al., 2010). Here, these P-granule proteins extend the permeability barrier of NPCs into the cytoplasm of germ cells (Updike et al., 2011). In the adult germline of *C. elegans*, loss of P granules causes sterility and germ cell reprogramming (Campbell and Updike, 2015; Knutson et al., 2017; Updike et al., 2014) suggesting that P granules maintain pluripotent potential by buffering the effects of inappropriate transcription in the germline, and that their FG-repeats are intrinsic to this role. In addition to FG-Nups and the five P-granule FG-repeat proteins above, FG-repeats are found in EGO-2, a regulator of GLP-1/Notch signaling in the germline, and in two undescribed proteins, K01A6.4 and PQN-75. EGO-2, K01A6.4, and PQN-75 are unique in that their FG-repeats are glutamine/asparagine (Q/N)-rich; however, it was not known if these three proteins function in or associate with germline NPCs or P granules.

Here we describe an allele of PQN-75 isolated from an EMS mutagenesis screen that prompted us to ask whether it regulates P-granule homeostasis. Interestingly, we discovered that PQN-75 is expressed in pharyngeal gland cells and is dispensable for germline development. Pharyngeal gland cells consist of five cells in the terminal (posterior) bulb of the pharynx that each extend anterior processes through muscle to contact points in the pharyngeal lumen (reviewed in Mango, 2007). Secretions from pharyngeal gland cell process have been observed just prior to hatching, suggesting they aid in cuticle digestion (Singh and Sulston, 1978). Pharyngeal gland cell specification requires the transcription factor HLH-6, and worms without HLH-6 or pharyngeal gland cells exhibit delayed growth and partially penetrant larval arrest (Smit et al., 2008).

HLH-6 is related to the mammalian salivary gland transcription factor Sng1; both mammalian salivary glands and gland cells in *C. elegans* secrete mucin-like proteins to aid in digestion, suggesting that these glands are evolutionarily related (Smit et al., 2008). In this study we describe similarities between PQN-75 and the human basic salivary proline-rich pre-protein PRB2, further strengthening this evolutionary relationship. We also explore the function of PQN-75 within pharyngeal gland cells by quantifying growth, larval development, and strain health in a *pqn-75* deletion strain, finding only subtle defects that potentially reflect a somewhat vestigial role of pharyngeal gland cells under laboratory growth conditions.

## RESULTS

**Fig. 1.**
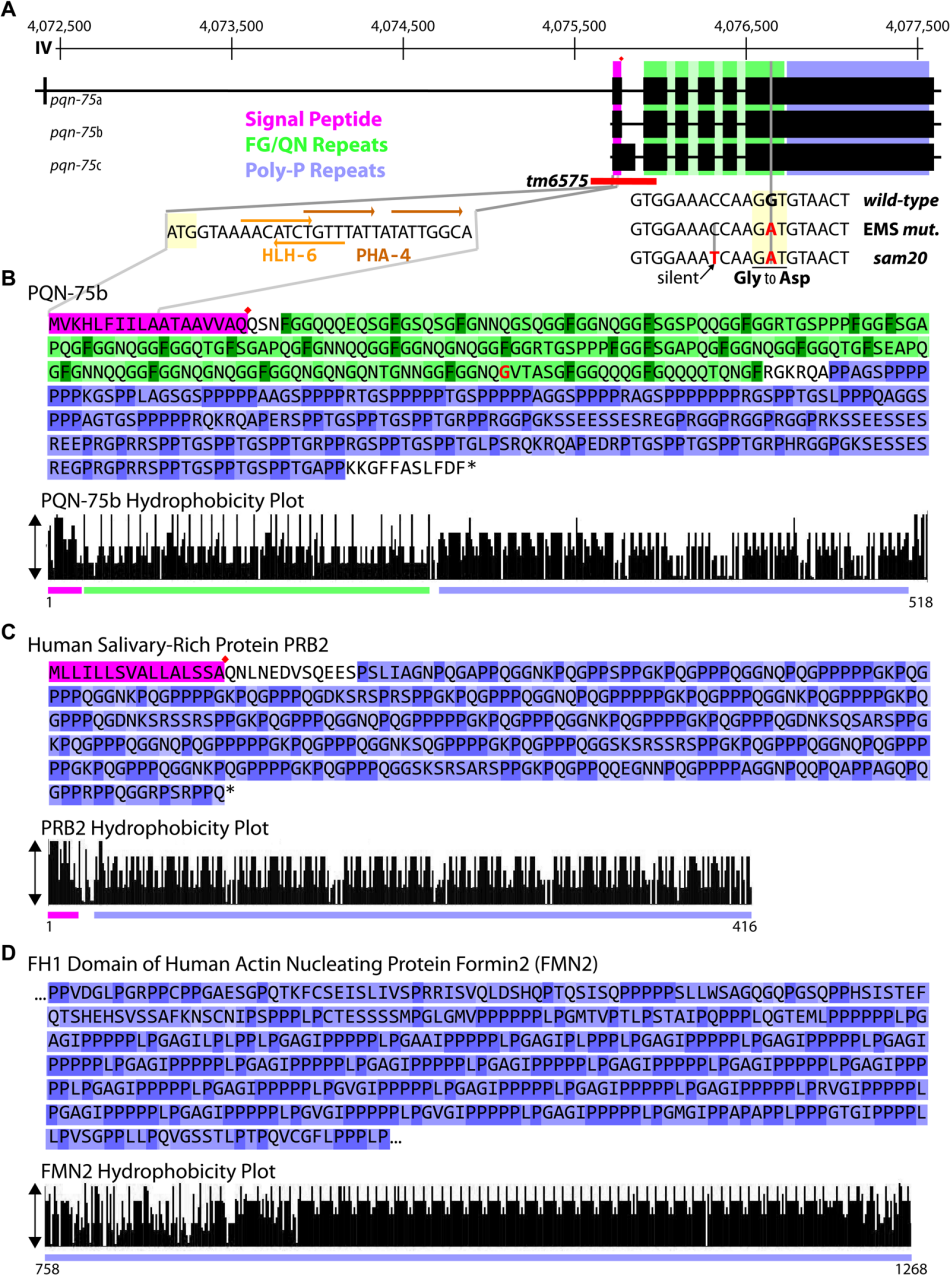
**PQN-75 protein domains.** (A) Three *pqn-75* isoforms are shown on chromosome IV, with their signal peptide (pink, red diamonds indicate position of predicted cut sites), FG/QN repeats (green), and polyproline repeat domains (blue) indicated. *pqn-75* alleles used in this study include *tm6575* (red bar), and the Gly to Asp mutations from EMS and *sam20* (reading frame indicated by a light yellow box). The coding sequence of N-terminal *pqn-75* contains putative HLH-6 and PHA-4 transcription factor binding sites for pharyngeal gland cell expression. (B) Location of the signal peptide, FG/QN and polyproline repeats in the PQN-75b sequence. A hydrophobicity plot (taller bars are more hydrophobic) demonstrates the regularity of FG (dark green) and QN (light green) and proline residues (dark blue) in PQN-75b. (C,D) Similarity of extended polyproline repeats in human PRB2 and FNM2 protein sequences.

A mutagenesis screen for effectors of P-granule assembly and distribution isolated multiple alleles of a gene encoding the Argonaute protein CSR-1 (Andralojc et al., 2017). Whole genome sequencing revealed that one of these alleles, *csr-1(sam18)*, also contained a linked Gly to Asp mutation in *pqn-75* (Fig. 1A). EMS mutagenesis introduces, on average, just over 300 variants per strain, approximately 50 of which alter or disrupt gene function (Flibotte et al., 2010); so linked mutations are not only common after outcrossing EMS generated alleles, but expected. PQN-75 is of particular interest because it contains domains common in a number of core P-granule proteins, necessitating a subsequent investigation into whether this new mutation is collateral or if it contributes to the P-granule phenotype of *sam18* worms.

### PQN-75 includes a unique combination of protein motifs

PQN-75 is an unusual protein. As its name suggests, the N-terminal half of PQN-75 contains a Q/N-rich ‘prion’ domain (Fig. 1A,B) (Michelitsch and Weissman, 2000). What distinguishes the Q/N-rich region of PQN-75 is that these polar residues are separated approximately every ten amino acids by a hydrophobic phenylalanine (F) flanked by glycine (G), generating cycles of regular hydrophobicity within this FG/QN repeat domain (Fig. 1B, green). In addition to the FG domain of PQN-75, three of its isoforms contain an N-terminal signal peptide (Fig. 1A,B, pink) with a predicted cleavage site (Fig. 1A,B, red diamond), suggesting that PQN-75 is a secreted protein. The C-terminal half is also unique in that it is proline-rich (i.e. 35% of the amino acids in the C-terminal half are prolines), primarily consisting of GSPP repeats (Fig. 1A,B, blue). While high proline content is indicative of a collagen-related structural protein, PQN-75 lacks cysteine residues important for cross-linking elongated collagen fibrils.

Clear PQN-75 orthologs exist in other *Caenorhabditis* species (*Caenorhabditis remanei*, *Caenorhabditis brenneri*, *Caenorhabditis briggsae*, and to a lesser extent in *Caenorhabditis japonica*) that contain the signal peptide, FG-repeat, and polyproline domains, but orthologs carrying all three of these domains are not apparent in the diplogastrid nematode *Pristionchus pacificus* or beyond. Wormbase (www.wormbase.org) lists the closest human homolog of PQN-75 as the human basic salivary proline-rich pre-protein PRB2 (e-value: 1.2e-45; % length: 54%). This secreted pre-protein has a signal peptide but lacks FG-repeats, and its function in the saliva is unknown (Fig. 1C). Another protein similar to PQN-75 that has both an N-terminal Q/N domain and a sizable C-terminal proline-rich repeat is human Formin-2 (e-value: 3.7e-31), a perinuclear actin-nucleating protein that confers nuclear integrity during cell migration (Skau et al., 2016). Unlike Formin-2 and the six Formin proteins in *C. elegans* (CYK-1, DAAM-1, FRL-1, FHOD-1, EXC-6, and INFT-2) (Mi-Mi et al., 2012), PQN-75 contains only the proline-rich Formin Homology domain one (FH1), but not FH2 or FH3 domains (Fig. 1D), making it unlikely that PQN-75 functions as a Formin.

### PQN-75 is dispensable for germline development

**Fig. 2.**
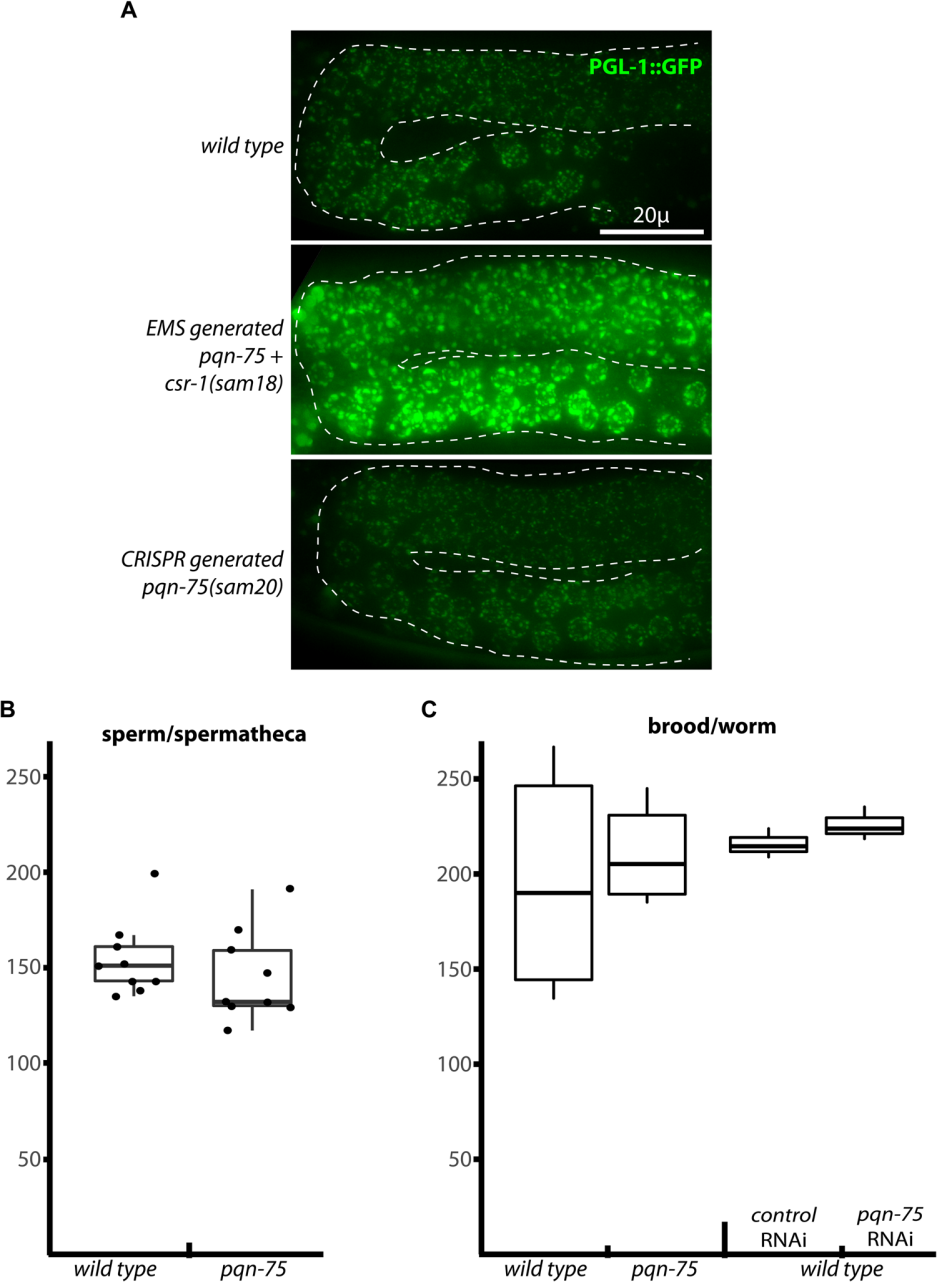
**Germline phenotypes of *pqn-75*.** (A) An EMS-generated Gly to Asp missense mutation in *pqn-75* does not contribute to the enlarged P-granule expression phenotype of *csr-1(sam18)* worms. Representative images show P granules surrounding germ cell nuclei navigating the bend in the gonad arm during the fourth larval stage. (B) DAPI-stained sperm nuclei/spermatheca in wild type and *pqn-75(tm6575)* mutants. (C) Brood size in wild type and *pqn-75(tm6575)* mutants, and in wild-type worms fed empty vector control or *pqn-75* RNAi. Box and whisker plots indicate the median, 1st and 3rd quartiles, and the minimum and maximum data points (excluding outliers - circles).

To determine the role of PQN-75 in the germline and whether the EMS-generated *pqn-75* allele affects P-granule size and distribution independent of *csr-1(sam18)*, these two linked mutations needed to be separated. This was done using CRISPR/Cas9 to recreate the single base pair mutation in *pqn-75* and included silent mutations to prevent Cas9 recleavage (Fig. 1A); this new allele, *pqn-75(sam20)*, was crossed into a P-granule reporter (PGL-1::GFP). P granules in *pqn-75(sam20)* appeared indistinguishable from wild-type worms, suggesting the original EMS-generated mutation was collateral and had no bearing on the P-granule phenotype of *csr-1(sam18)* (Fig. 2A). Sperm counts were compared in the predicted *pqn-75* null allele *tm6575* (see Fig. 1A, red bar) and no appreciable difference was found (Fig. 2B). Brood sizes in the *pqn-75* mutant and with *pqn-75*(RNAi) were just as high as controls (Fig. 2C), suggesting PQN-75 does not impact fertility and plays little or no role in the germline.

### PQN-75 is expressed in pharyngeal gland cells

**Fig. 3.**
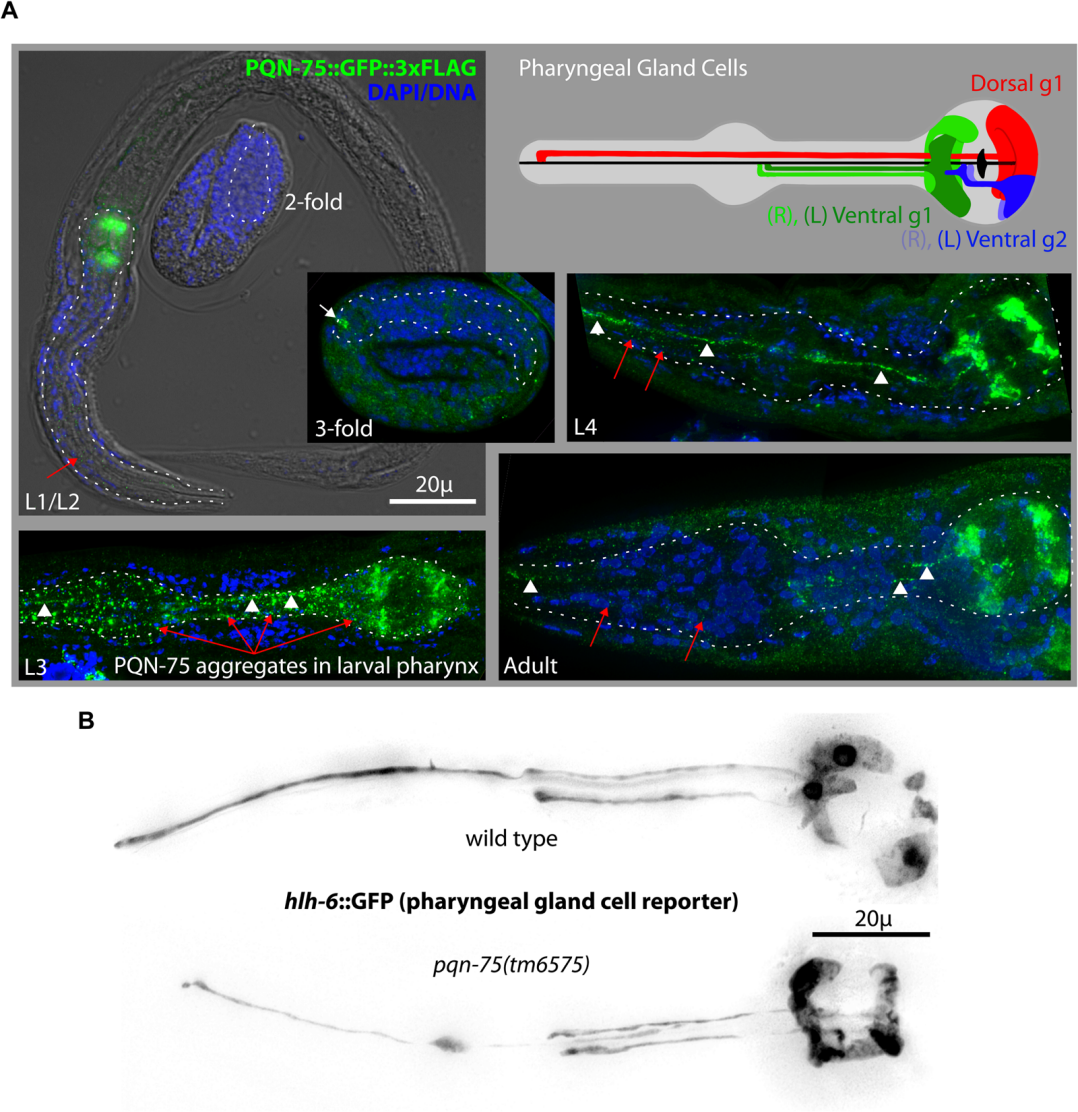
**PQN-75 expression.** (A) Cartoon (adapted from http://wormatlas.org/) shows the five pharyngeal gland cells extending processes into the anterior (red), mid (green), and posterior (blue) pharyngeal lumen (black). In fixed worms, anti-FLAG staining of PQN-75 (green) first appears in the threefold stage of embryogenesis (arrow) and PQN-75 aggregates persist in the pharyngeal gland cells and their processes (arrowheads) through larval stages and in the adult, and are frequently found throughout pharyngeal muscle (red arrows). Dotted lines outline the pharynx of each worm. (B) Images of *hlh-6::*GFP expression in wild-type and *pqn-75(tm6575)* animals.

Several FG-repeat containing proteins (e.g. GLH-1, GLH-2, GLH-4, DDX-19, and RDE-12) are enriched in germline P granules (Gruidl et al., 1996; Sheth et al., 2010); however, expression profiling suggests that PQN-75 may not share this subcellular localization as its transcripts are minimally expressed in dissected germlines (0.3 FPKM; 12,155th of 20,259 genes ranked by germline expression) (Campbell and Updike, 2015). Lines carrying fluorescent *pqn-75* reporters are available, showing expression in the terminal bulb of the pharynx but not the germline (Mounsey et al., 2002). Since the germline frequently silences repetitive reporters, CRISPR was used to tag *pqn-75* with GFP::3xFLAG so endogenous gene expression in the germline could be examined. Again, extremely faint expression was only observed in the posterior pharynx. To amplify the PQN-75::GFP::3xFLAG signal, worms were fixed and stained green with M2 anti-flag and a blue DAPI/DNA costain, and still there was no evidence of germline expression (Fig. 3A). PQN-75 staining was exclusively in the pharynx, starting in the threefold stage of embryogenesis (arrow), becoming progressively more pronounced through larval development. Within the pharynx, PQN-75 was most abundant in the pharyngeal gland cells and could be observed in gland-cell processes that extend along the pharyngeal lumen (Fig. 3A, arrowheads). Poly Q/N and FG repeats have the propensity to promote self-assembly and aggregation in a number of proteins; similarly, punctate PQN-75 aggregates are found in the processes and pharyngeal gland cell bodies.

Pharyngeal gland cell function is heavily inferred from cell shape, position, and gene expression. It is thought that pharyngeal gland cell secretions lubricate the pharyngeal lumen and aid in molting or formation of the surface coat on the anterior cuticle (reviewed in Pilon, 2014). While worms are still viable following genetic ablation of gland cells, they exhibit delayed growth, development, and partially penetrant larval arrest (Smit et al., 2008). Interestingly, PQN-75 staining was not detected within the pharyngeal lumen, buccal cavity, or on the anterior cuticle. Instead, 77% (*n*=200) of larval-staged worms had varying amounts of PQN-75 aggregates throughout the pharynx, suggesting that gland cells secrete PQN-75 into the surrounding pharyngeal muscle (Fig. 3A, red arrows). This is in contrast to the recently described abu/pqn paralog group (APPG) genes that encode poly Q/N proteins in pharyngeal muscle, which are excreted to form the anterior cuticle (George-Raizen et al., 2014). PQN-75 also differs from mucin-like PHAT-5, which is secreted from pharyngeal gland cells to line the pharyngeal lumen (Smit et al., 2008).

Pharyngeal gland cell expression is enacted through combinatorial signaling of the sequence-specific transcription factors PHA-4 and HLH-6 (Gaudet and Mango, 2002; Raharjo and Gaudet, 2007). Correspondingly a short 22 base pair sequence at the beginning of the PQN-75 coding region contains two tandem PHA-4 consensus binding sites (TRTTKRY) and an HLH-6 consensus binding site palindrome (AACANNTGTT) that may promote gland cell expression of PQN-75 (Fig. 1A). To determine if PQN-75 is required to drive pharyngeal gland cell specification or morphology, *hlh-6*::YFP arrays were introduced in wild type and *pqn-75(tm6975)* mutants to light up pharyngeal gland cells and their processes (Fig. 3B). All five pharyngeal gland cells were present in wild type and *pqn-75* mutants, and no differences in cell morphology or process extension could be distinguished between the two strains throughout larval development and in adults (>30 worms imaged for each strain). This suggests that PQN-75 is not required for gland cell survival or morphology.

**Fig. 4.**
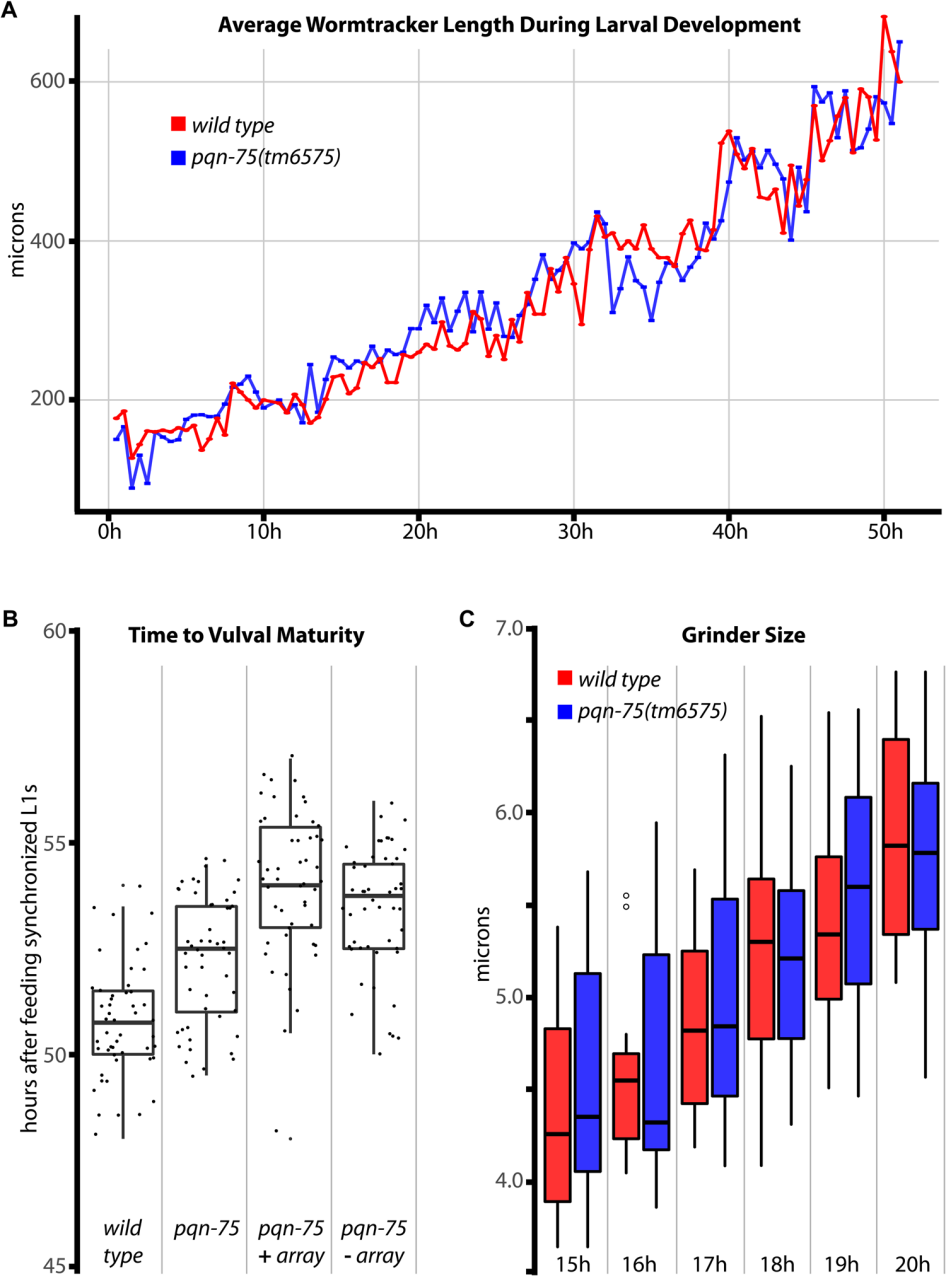
**Larval development of *pqn-75*.** (A) Wormlab software was used to capture the collective average length of wild-type and *pqn-75* worms (*n*>30 worms/time point) every half hour for the first 51 h of larval development. (B) Time to vulval maturity in wild type, *pqn-75* mutants, *pqn-75* mutants rescued with a wild-type *pqn-75* array, and in the rescued worms after losing the array. (C) Grinder width of wild type and *pqn-75* mutants (*n*=20 worms/time point). Box and whisker plots indicate the median, 1st and 3rd quartiles, and the minimum and maximum data points (excluding outliers - circles).

Pharyngeal gland cell expression may implicate a role for PQN-75 in feeding, digestion, or molting, all of which should be reflected in the growth rate. To test this, *pqn-75(tm6575)* and *wild-type* L1 worms were synchronized, and growth and time to sexual maturity were compared. Worm length was measured in approximately 30 worms every hour for 52 h using automated worm-tracking software, but no difference could be observed between the two strains (Fig. 4A). Sexual maturity was measured by the time to reach the young adult stage as marked by vulval maturation. While *pqn-75* mutants were delayed 1.5 h (*P*<1×10^−6^), worms carrying *pqn-75* fosmid arrays did not rescue this delay in the mutant, suggesting this minor delay could be attributed to possible background mutations (Fig. 4B). Obvious molting phenotypes were not apparent in *pqn-75(tm6575)*. To detect more subtle effects on growth and molting, the width of the grinder was measured as it grows in a salutatory fashion during each molt (George-Raizen et al., 2014). For each strain, grinder width was measured in 15 worms from synchronized cultures between 15 and 20 h to capture the window of the L1 to L2 molt (Fig. 4C). Grinder width was comparable in wild type and *pqn-75(tm6575)* mutants, suggesting that PQN-75 has no significant or detectible role in molting.

### PQN-75 promotes thermotolerance, but minimally impacts innate immunity and proteotoxic stress

**Fig. 5.**
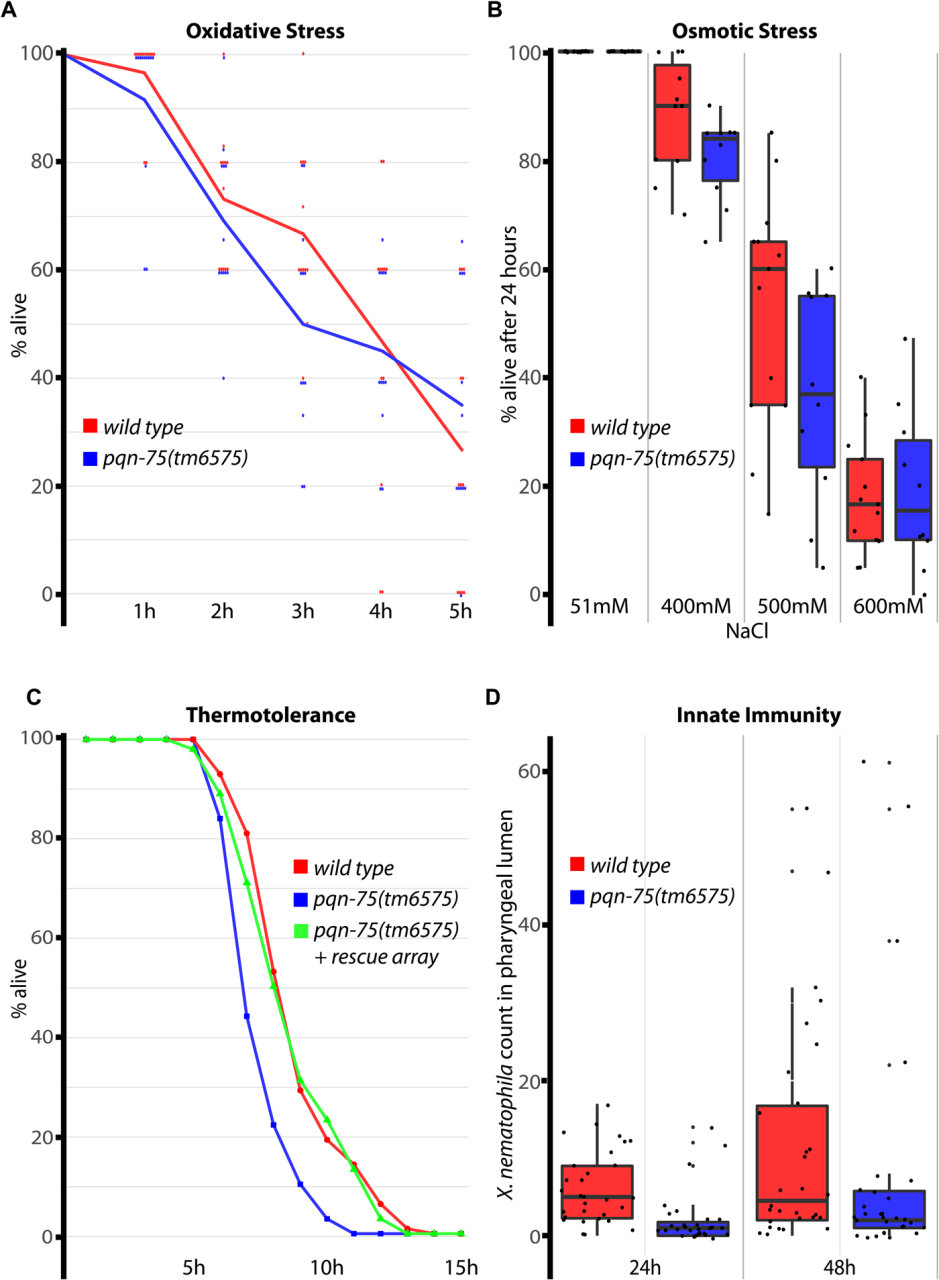
**Proteotoxic stress and immunity in *pqn-75*.** (A) Oxidative stress was induced with Paraquat, and viability was monitored over a five-hour period. Data points are included for all 12 replicates of each strain. (B) Osmotic stress was induced by exposure to high salt, and viability was scored after 24 h. (C) Heat stress was induced by growth at 37°C, and viability was monitored each hour for 15 h. (D) Innate immunity is measured by the accumulation of GFP-expressing *X. nematophila* in the pharyngeal lumen at 24 and 48 h. Box and whisker plots indicate the median, 1st and 3rd quartiles, and the minimum and maximum data points (excluding outliers).

Optimal conditions in the laboratory will often mask subtle defects caused by mutated genes. Proteins with Q/N prion-like domains, like those found in PQN-75, have a propensity to aggregate, which could burden cellular protein homeostasis machinery (Moronetti Mazzeo et al., 2012). To test whether PQN-75 impacts homeostasis, *pqn-75(tm6575)* worms were challenged and their response to various forms of stress recorded. First, paraquat was used to induce oxidation/glutathione conjugation of proteins; no survival advantage or disadvantage was conferred after five hours of exposure by the presence of PQN-75 (Fig. 5A). Second, osmotic stress was used to induce protein misfolding, and while there was a trend for survival rates of *pqn-75* mutants to be lower after 24 h of growth in hyperosmotic environments, it was not significant (Fig. 5B, *P*>0.05). Third, protein misfolding was induced with heat stress. In this case, *pqn-75(tm6575)* viability decreased more rapidly than wild type when grown at 37°C (Fig. 5C, *P*<0.001, log rank). To test specificity, thermotolerance was observed in *pqn-75* mutant worms carrying an array with wild-type *pqn-75* sequence, which rescued survivability (*P*<0.001). These results suggest that the presence of PQN-75's Q/N prion-like domains do not exacerbate proteotoxic stress; instead, the presence of PQN-75 confers some advantage when worms are stressed, especially upon exposure to high temperatures.

Because the surface coat of the anterior cuticle provides innate immunity against pathogen biofilm and colonization, wild type and *pqn-75* mutants were compared on plates seeded with the bacteria *Xenorhabdus nematophila*. *X. nematophila* exists in a symbiotic relationship with soil nematodes that parasitize insects, and when fed to *C. elegans* it can produce a biofilm on the cuticle and in the lumen of the pharynx (Couillault and Ewbank, 2002). *X. nematophila* expressing green fluorescent protein (GFP) was fed to both wild-type and mutant worms, and the number of bacteria in the pharyngeal lumen was quantified at 24 and 48 h (Fig. 5D). The pharyngeal lumen of *pqn-75* mutants was not more impacted than wild type; in fact, mutants had slightly fewer bacteria in the lumen at 24 h (6.2 bacteria in wild type versus 2.1 in *pqn-75*, *P*<1×10^−3^) and 48 h (11.6 versus 7.9, *P*=0.35), raising the possibility that PQN-75 negatively impacts innate immunity. This difference may be insubstantial, and the response to a larger panel of natural pathogens would be warranted before reaching a conclusion about PQN-75's role in innate immunity.

## DISCUSSION

Unlike the majority of FG-repeat proteins, which associate with the nuclear pore complex or germline P granules, PQN-75 aggregates disperse throughout the cytosol of pharyngeal gland cells and appear to be secreted into surrounding pharyngeal muscle, although the function of PQN-75 aggregates in these cells remains unclear. We have demonstrated that PQN-75 does not affect brood size or growth rate, and minimally impacts osmotic stress and innate immunity. Yet, *pqn-75* mutants are more susceptible to heat stress, suggesting that PQN-75 protects the organism from acute temperature changes.

The surface coat of the anterior cuticle is thought to originate from pharyngeal gland cell secretions, and the mucin-like gland cell protein PHAT-5 associates with the cuticle when secreted (Smit et al., 2008). While PQN-75 was not found on the cuticle or in the pharyngeal lumen, detection relied on a C-terminal GFP::3xFLAG tag on the endogenous gene and secretions would not be observed if the protein is subject to proteolysis, and there may be precedence for this. The human salivary proline-rich-proteins (PRPs) are the most conserved oral salivary proteins among mammals, representing 20-30% of proteins in saliva, but their function remains unknown (Manconi et al., 2016). Humans have six salivary PRPs that cluster on chromosome 12, and each undergoes proteolysis into much smaller peptides. PRB2 (Fig. 1C) is one of these processed pre-proteins containing polyproline repeats like PQN-75. Both worm PQN-75 and human PRB2 are expressed in gland cells of the upper digestive tract; therefore, one possibility is that PQN-75 also undergoes proteolysis, preventing the detection of N-terminal secretions in the lumen or cuticle with the C-terminal tag. If secreted to the lumen or cuticle, one could predict that PQN-75 promotes the digestion or prevents the pathogenesis of bacteria regularly encountered in its natural environment, or alternatively contributes to the structural integrity of the cuticle, making the worms less susceptible to fluctuations in temperature.

If the primary function of PQN-75 is within the gland cell itself, the propensity of PQN-75 to self-aggregate may be nucleating cytoskeletal structure. Polyproline helixes are common in both globular and structural proteins and frequently serve as the interface between protein-protein interactions (Manconi et al., 2016). The long polyproline repeat (FH1 domain) of Formin-2 (Fig. 1D) shares similarities with the polyproline domain of PQN-75. Formins use their FH1 domain to interface with profilin to nucleate actin polymerization (Schönichen and Geyer, 2010), and interestingly Formin-2 (FMN2) specifically nucleates actin filaments on the nucleus to maintain nuclear shape and integrity during cell migration (Skau et al., 2016). Of the six formins expressed in *C. elegans*, three of them (EXC-6, INFT-2, and CYK-1) are expressed in the large excretory cell, which forms tubules along the length of the worm and functions in osmoregulation (Shaye and Greenwald, 2016). In the excretory cell, these three formins polymerize actin to regulate tubulogenesis. Outside of the excretory cell, the formins CYK-1 and FHOD-1 associate with body-wall muscle sarcomere Z lines to promote muscle contractility (Mi-Mi et al., 2012).

Pharyngeal gland cells do not undergo tubulogenesis, but their long processes are like those of the excretory cell and may also require profilin-dependent actin nucleation during elongation. Alternatively, PQN-75 secreted into the surrounding pharyngeal muscle may stimulate muscle contractility. We used this reasoning to look for genetic interactions between *pqn-75* and the three profilins in *C. elegans* (*pfn-1*, *pfn-2*, and *pfn-3*) but did not find signs of enhanced lethality, sterility, or growth defects. PQN-75 appears not to affect pharyngeal gland cell process extension as viewed by the *hlh-6*::YFP reporter, so a clear role for this protein and actin polymerization in pharyngeal gland cells has yet to be determined.

Many genes with unknown functions (40% in *C. elegans*) are likely important in natural settings (Petersen et al., 2015). *pqn-75* is one of these genes, and its growth under laboratory conditions may mask its functions, such as PQN-75's protective effect at higher temperatures. It is also worth noting that *pqn-75* expression increases in response to dietary restriction and pathogen exposure (Baugh et al., 2009; Engelmann et al., 2011; Heestand et al., 2013; Mueller et al., 2014; Yang et al., 2015; Jarod Rollins and Aric Rogers, personal communication), further suggesting a role for PQN-75 in feeding and innate immunity. Once laboratory growth conditions better reflect the worm's natural diet and ecology, additional *pqn-75* phenotypes will become apparent to clarify this gene's function.

## MATERIALS AND METHODS

### Strain maintenance

*C. elegans* strains were maintained in accordance to standard protocol (Brenner, 1974). The following stains were obtained through the *Caenorhabditis* Genetics Center (CGC): N2(Bristol), RW11454 *stIs11454[pqn-75b::H1-wCherry+unc-119(+)].* The deletion allele TM6575 *pqn-75(tm6575)* was obtained from the National Bioresource Project in Japan, 3× outcrossed, and sequenced to confirm the annotated deletion was present and homozygous. The following strains were created for this study, and are available upon request: DUP17 *ddEx16[pgl-1p::PGL-1::TY1::EGFP::3XFLAG(92C12)+Cb-unc-119(+)]* I, DUP36 *pqn-75(EMS) csr-1(sam18)IV/nT1[qIs51](IV;V); ddEx16[pgl-1p::PGL-1::TY1::EGFP::3XFLAG(92C12)+Cb-unc-119(+)]* *I*, DUP38 *pqn-75(sam20) IV, DUP49 ddEx16[pgl-1p::PGL-1::TY1::EGFP::3XFLAG(92C12)+Cb-unc-119(+)]* I; *pqn-75(sam20)* IV, DUP 116 *pqn-75(tm6575)* IV*;* *samEx7(pqn-75 fosmid WRM0639dH02+pCFJ104),* DUP66 *pqn-75[sam26(pqn-75::GFP::3xFLAG)]* IV, DUP129 *samEx9(hlh-6::YFP+myo-3::mCh); pqn-75(tm6575)* IV.

### CRISPR strain construction

To recreate the G to A base pair change in the *sam18* allele of *pqn-75*, plus silent mutation in the PA motif to prevent recleavage, the sgRNA (AATCCGCTAGCAGTTACACCT) was used to make the Cas9/sgRNA plasmid pDU54 and coinjected with a 60 bp HR oligo (TTGGCCTCCGAATCCGCTAGCAGTTACA**T**CTTG**A**TTTCCACCGAATCCTCCATTGTTTCC) using the *rol-6* Co-CRISPR method (Ward, 2014). Bold letters indicate changes in the sequence. Edits were sequence confirmed and homozygosed.

Endogenous *pqn-75* was tagged with GFP::3xFLAG using the FP-SEC method (Dickinson et al., 2015), where the sgRNA (GCGAAGAATCCCTTCTTTGG) was used to make the Cas9/sgRNA plasmid pDU61, and coinjected with a GFP-SEC flanked with *pqn-75* sequence (pDU66) to make the C-terminal insertion with 10 silent mutations to prevent Cas9 recleavage. The expected edits were sequence confirmed and homozygosed.

### Analysis of germline integrity

Synchronized L4-staged DUP17, DUP36 (non-green pharynx), and DUP49 worms were live mounted on agarose slides. Images of the bend in the gonad were acquired for each strain using fixed exposure conditions. 60× objective on a Leica DMI6000B – 10 gonad arms/strain.

To count sperm, synchronized young adults were fixed in M9 with 8% PFA for 1 h, washed 3× with PBS, 1× with 95% ethanol for 1 min, 3× with PBS then mounted on a charged slide with mounting media containing DAPI. Sperm nuclei were imaged and counted in each spermatheca using a 60× objective on a Leica DMI6000B – 10 worms/strain.

To count brood size, six L4 stage worms were picked to a plate and passaged to new plates each day for six days. The number of progeny were totaled from each plate and divided by six. This was repeated three times to get the average broods of N2 versus *pqn-75(tm6575)* and N2 fed control (empty vector) versus *pqn-75* RNAi. RNAi feeding was performed as previously described (Kamath et al., 2001).

### Immunostaining

PQN-75::GFP::3xFLAG worms were fixed using methanol/acetone+DAPI (Strome and Wood, 1983). Antibody dilutions were 1:1000 M2-anti-FLAG (Sigma Aldrich), and 1:500 Goat anti-mouse Alexa Fluor 488 (ThermoFisher). Images were acquired using a 60× objective on a Leica DMI6000B and deconvolved with Leica imaging software.

### Worm growth assays

100-200 synchronized N2 and *pqn-75(tm6575)* L1's were plated onto the OP50 lawn. Worm Tracker (Micro Bright Field Inc. Stable camera stand with WormLab software and ATV Stingray F504B camera attached to Nikon macro lens) was used to monitor worm length every half hour for 51 h. Captured video was 30 s in length using a section of the plate within the bacterial lawn, quantifying worm length for 10-60 worms for each time point. Lengths captured are slightly shorter than normal due to the program's inability to accurately capture the small diameter of the worm's tail. A collective average was used to plot the lengths.

Vulval development was monitored by synchronizing L1 larvae and plating 50/plate at time zero. After 48 h of growth at 20°C, worms were removed from the plate when a fully developed vulva was evident. Observation ended when all worms reached full vulval development.

To measure grinder development, synchronized L1s were placed on food and imaged each hour from 15-20 h after feeding. At each time point, 20 worms from each strain were placed in 5 mM levamisole on an agar slide, and the width of their grinder was measured using a 40× DIC objective and Leica imaging software (George-Raizen et al., 2014).

### Proteotoxic stress assays

Oxidative stress was induced in L4 worms by exposure to 100 mM Paraquat in a 96-well plate assay (10 worms/well, 12 replicates/strain) and survival was scored every hour as described (Possik and Pause, 2015).

Osmotic stress was induced in L4 worms upon growing on seeded NGM plates containing 51 mM NaCl (normal NGM levels), and higher NaCl concentrations of 400 mM, 500 mM, and 600 mM, and survival was scored 24 h later as described (Lee et al., 2016).

Heat stress was induced in synchronized young adult worms (2 plates, 50 worms/plate/strain) by incubating them at 37°C. Plates were checked each hour, and dead worms (not responding to physical stimulation and no pharyngeal pumping) were removed from the plate.

### Biofilm formation

Synchronized *pqn-75(tm6575)* and wild-type worms were plated on GFP-expressing *X. nematophila* ATCC19061-007-GFP bacteria. At 24 and 48 h, worms were resuspended for five minutes 1× Egg Buffer (118 mM NaCl, 448 mM KCl, 2 mM CaCl2, 2 mM MgCl2, 25 mM HEPES) with 1 mM levamisole, mounted on an agar slide, and imaged on a Leica DMI6000B. Green bacteria were counted in the pharyngeal lumen of 30 worms for each strain at each time point.
